# Design Preferences for a Serious Game–Based Cognitive Assessment of Older Adults in Prison: Thematic Analysis

**DOI:** 10.2196/45467

**Published:** 2023-04-17

**Authors:** Rhys Mantell, Adrienne Withall, Kylie Radford, Michael Kasumovic, Lauren Monds, Ye In Jane Hwang

**Affiliations:** 1 School of Population Health University of New South Wales Sydney Australia; 2 Ageing Futures Institute University of New South Wales Sydney Australia; 3 School of Psychology University of New South Wales Sydney Australia; 4 Neuroscience Research Australia Sydney Australia; 5 School of Biological, Earth & Environmental Sciences University of New South Wales Sydney Australia; 6 Arludo Sydney Australia; 7 Addiction Medicine, Central Clinical School The University of Sydney Sydney Australia

**Keywords:** serious game, gamification, cognitive assessment, prison, older adults, older prisoners, game design, self-determination theory

## Abstract

**Background:**

Serious games have the potential to transform the field of cognitive assessment. The use of serious game–based cognitive assessments in prison environments is particularly exciting. This is because interventions are urgently needed to address the rapid increase in the number of currently incarcerated older adults globally and because of the heightened risks of dementia and cognitive decline present in this population. Game-based assessments are assumed to be fun, engaging, and suitable alternatives to traditional cognitive testing, but these assumptions remain mostly untested in older adults. This is especially true for older adults in prison, whose preferences and needs are seldom heard and may deviate from those previously captured in studies on cognition and serious games.

**Objective:**

This study aimed to understand the design preferences of older adults in prison for a game-based cognitive assessment.

**Methods:**

This study used reflexive thematic analysis, underpinned by critical realism, and applied the technique of abduction. Overall, 4 focus groups with a total of 20 participants were conducted with older adults (aged ≥50 years; aged ≥45 years for Aboriginal and Torres Strait Islander people) across 3 distinct prison environments in Australia.

**Results:**

Self-determination theory was used as a theoretical foundation to interpret the results. Overall, 3 themes were generated: *Goldilocks*—getting gameplay difficulty *just right* through optimal challenge (the first theme emphasizes the participants’ collective desire for an individualized optimal level of difficulty in serious gameplay), *Avoiding Childish Graphics*—gimmicky gameplay can be condescending (the second theme raises the importance of avoiding immature and childlike gameplay features, as some older end users in prison felt that these can be condescending), and *A Balanced Diet*—meaningful choice and variety keeps game-based assessments fun (the third theme highlights the strong user preference for meaningful choice and variety in any serious game–based cognitive assessment to maximize in-game autonomy).

**Conclusions:**

The collection of these themes provides novel insights into key game design preferences of marginalized older adults.

## Introduction

### Assessing Cognition Through Serious Games

Gamification stands as one of the most promising solutions to enhance the user experience of traditionally monotonous tasks [1]. The process of gamification includes applying game design elements such as challenges, scoring, graphics, and narratives to nongame environments (eg, psychometric testing) to increase engagement and motivation [2]. When gamified tasks are embedded into an immersive user experience, they are typically referred to as *serious* games. Serious games present users with unique and exciting environments to complete objectives that go beyond entertainment [3]. An emerging area of research is the study of serious games for cognitive assessment. Although there are well-evidenced benefits of using traditional cognitive assessment tools (proven psychometric validity, high sensitivity to neurological disease, etc) [4-6], they are often seen as boring and repetitive by users [7]. This can reduce task engagement [2] and affect the reliability of the performance data collected [1,8]. People also report test anxiety and self-stigma regarding low literacy or education levels when performing traditional cognitive tests [9-12]. In addition, there is evidence that some widely used neuropsychological tests are not culturally safe [13-15].

Serious games have the potential to transform the field of cognitive assessment. Game-based assessments appear to be suitable alternatives to traditional tasks given their potential to improve motivation and enjoyment and support individuals to produce their best effort, without making them feel overly bored, anxious, or distracted [1,16]. Recent studies have indicated that the experience of playing digital cognitive games was perceived as less stressful, more interesting, and more enjoyable when compared with standard cognitive tasks [2,3]. Serious games can also offer a brief, cost-effective, and scalable substitute for more traditional assessment methods [8,17]. In addition, serious games might support more ecologically valid assessment through realistic context and gameplay by engaging cognitive processes in a way that is more similar to a real-life situation [18].

Although the user benefits of enhanced motivation, engagement, and enjoyment are promising, they are not automatically derived through gamification. In recent years, some gamified cognitive assessments have assumed that, because something is presented as a game, it is fun. In a systematic review (conducted in 2021) of game-based cognitive assessments [1], less than half of the games included in the study evaluated any aspect of gameplay or user preference. Furthermore, although many studies have reported the positive effects of serious games on enjoyment [8], additional findings suggest that some games do not increase engagement, with Birk et al [19] concluding that their gamification actually reduced task engagement when compared with a traditional task. These findings suggest that serious games that assume user enjoyment and engagement overlook the complexity of end-user preferences. Poorly designed and executed serious games can be expensive and ineffectual and are unlikely to result in a more enjoyable and motivating experience than traditional tasks [1,16,19,20]. If games are to be used as cognitive assessment tools, they must first be designed with the end user’s enjoyment and motivation in mind. Furthermore, ensuring that a game-based assessment is culturally safe to play is critical [21]. This means acknowledging the context in which a serious game will be played by a user and ensuring a relevant level of sensitivity to the distinct backgrounds, beliefs, and experiences of target users [21]. These are essential steps in justifying the use of a serious game, especially in the context of cognitive assessment.

### The Cognitive Health of Older Adults in Prison

There has been a rapid increase in the number of people aging in prisons across the world in the last decade [22]. One area of particular concern for older adults in prison is age-related cognitive decline. There is evidence indicating that cognitive deficits are considerably underdetected in prison environments [23], and when they are detected, they are often managed informally or inadequately [24,25]. Studies on cognitive impairment in Australia, France, and the United Kingdom reported high levels of potential cognitive decline, with impairment rates of 15.3% (Australia) [26], 19% (France) [27], and 13% (the United Kingdom) [28] found in samples of people aged ≥50 years (≥45 years for Australian Aboriginal and Torres Strait Islander people) in prison.

Cognitive impairments lead to poor outcomes for older adults in the criminal justice system [23,29] and those transitioning out of prison [30]. Unidentified cognitive impairment can hamper a person’s ability to navigate the prison system and reduce their capacity to access (and routinely engage with) health and aged care services during incarceration and after being released from prison [31]. People with cognitive impairment are also prone to manipulation and coercion in prison, including physical and sexual trauma, peer pressure, and victimization [32]. People with cognitive impairments in prison can also have their symptoms perceived by staff and peers as purposefully obstructive or combative, resulting in punishment [33,34]. In addition, after being released from prison, people who have substantial cognitive impairments are less likely to adapt to *life on the outside* without appropriate support [24,25], resulting in increased risks of recidivism, homelessness, and hospitalization [30].

Although prison staff view early screening and diagnosis of dementia as a priority area for prison dementia care reforms [23,29,35], there still exists very limited cognitive assessment of older adults in prison, often because it is resource intensive. The use of a serious game for cognitive screening in prison appears to be a potential solution to the growing need for an effective and affordable cognitive assessment to support older adults in prison presenting with or at risk of cognitive deficits.

### Conceptualizing Motivation to Play Games in Prison Using Self-determination Theory

If gamification promises to enhance user motivation to undertake cognitive assessment, but gamification does not always fulfill this promise, then a way to understand what increases (or decreases) a person’s motivation is necessary. A prominent theory used to understand a person’s motivation is self-determination theory (SDT). In SDT, the basic needs that ought to be satisfied to produce motivation, enjoyment, and overall well-being are competence (ie, experiencing mastery over challenges), autonomy (ie, doing something owing to an individual’s own volition), and relatedness (ie, experiencing meaningful social relations). In addition to helping researchers understand motivation generally, SDT has also been applied specifically to understand the motivation to play digital games [36-38]. The theory has been useful in explaining how game features relate to basic psychological needs and thus how game design and development decisions can increase (or decrease) user motivation and enjoyment [39]. Games have been shown to directly enhance motivation through features and gameplay [38].

In the video game context, competence refers to being challenged at the optimal level. For instance, a player will experience low competence if the challenges are very great (eg, game controls are difficult to use or enemies are very numerous) [37]. Autonomy may include the feeling of free choice to play a desired game and make choices within the game (eg, choosing which level to complete). Autonomy may also be satisfied when a user can design their own playable character and feel empowered to do things that they cannot do in real life. Relatedness can be achieved by playing with others, either in person or via the web [36]. Relatedness or a sense of belonging or connection can be satisfied by achieving team goals (eg, defeating the enemy together) or through healthy competition (eg, racing each other in Mario Kart).

SDT is a helpful framework to understand the motivation to play a serious game–based cognitive assessment in prison. Specifically, it provides a theoretical foundation to understand a specific user group’s fundamental psychological needs [40-42] and map these needs to tailored game design preferences [37,38]. In doing so, it ensures that a game is designed appropriately with the end user’s enjoyment, motivation, and well-being in mind [39]. Using SDT to query game design preferences and acceptability is an important exercise capable of expanding knowledge about desired user preferences for a serious game in prison.

### Objectives of This Study

A critical first and ongoing step in developing suitable game-based assessment is to collaborate with the target population for whom the intervention is intended [43-45]. This point is especially important for older adults in prison whose preferences, values, and needs may deviate from those previously captured in studies on cognition and game design [46,47]. It is essential to capture and embed end-user input into game design and development to produce a game that is relevant to and compatible with the lives of older adults who are in prison.

Thus, the purpose of this qualitative thematic analysis was to understand the design preferences for a cognitive game-based assessment for older adults in prison, in the context of the prison environment and future transition back to community. Using the theoretical framework provided by SDT, the user design preferences for and user acceptability of a serious game–based assessment can be hopefully understood in a way that is useful for game designers to develop a cognitive assessment that is motivating, culturally suitable, nonthreatening, and enjoyable.

## Methods

### Philosophical Approach

This qualitative study was informed by a critical realist philosophy [48-51]. Critical realism broadly assumes that there are things that have a real, objective existence *out there* (ie, an independent world exists beyond our own constructions). However, critical realism appreciates that knowledge is fallible; our understanding of the world is based on and filtered through our own personalized interpretations [52]. Thus, critical realism makes an important distinction between epistemological and ontological assumptions. That is, ontology (ie, what is real and the nature of reality) is not reducible to epistemology (ie, our knowledge of reality) [48]. In other words, the critical realist approach combines realist ontology with constructivist epistemology [52]. After assuming this position, we can make ontological distinctions between our unique experiences and observations of events, the actual events themselves (which can be different from our experience of them), and the underlying mechanisms or structures that produce events [53]. Once we perceive reality in this way, we are open to exploring and explaining social events through reference to these mechanisms and structures and their effects.

Using the philosophical position of critical realism for this study has 2 major benefits. First, the deeply individual and subjective game preferences of older adults in prison can be interpreted empirically. Such an exercise can (and should) have direct input into game design considerations regarding the features, preferences, and esthetics of the game development process. Second, a more theoretical and critical analysis of participants’ observations and how the underlying mechanisms and structures may explain game preferences (and motivation for game-based cognitive assessments) can be undertaken. This second type of analysis is necessary to explore latent themes that may influence the success of a serious game–based intervention in the context of this complex population [54,55]. Considering both empirical observations and underlying mechanisms, simultaneously, and how they interact is critical to adequately design, develop, and deliver a game-based cognitive assessment for this marginalized population.

### Sampling and Recruitment

Purposive sampling was used to produce representation across age, sex, and security level. Correctional staff identified and recruited potential participants after guidance from researchers to identify any older adults in prison, that is, aged ≥50 years (or ≥45 years for Aboriginal and Torres Strait Islander people). Aboriginal and Torres Strait Islander people are the Indigenous people of Australia, and relatively young ages are typically used in health, aged care, and policy settings to define *older adults* in this population, owing to population-level disparities in morbidity and mortality. Suitable participants were known to corrective staff through their day-to-day operations. Staff oversampled those who were potentially eligible for the study and shared the study information (study flyers and consent forms) with them. Consent forms were signed by participants in front of the prison staff. Potential participants were allowed to ask any clarifying questions to staff. After the consent forms were signed, these were sent back to the research team for confirmation of informed consent.

We are sympathetic with the criticism by Braun and Clarke [56] about saturation as a useful concept in reflexive thematic analysis underpinned by constructionist epistemology. As such, we do not discuss our sample in terms of saturation. However, given our commitments to critical realist ontology, it is important to confirm the sufficiency of our sample in terms of information power [57] to support validity claims [52]. Thus, according to the criteria stipulated by Malterud et al [57], our sample size of 20 participants across 4 focus groups (FGs) was deemed sufficiently powerful given the (1) applied aim of the study, (2) sample specificity, (3) use of established theory (eg, SDT), (4) high quality of dialogue, and (5) comprehensive analysis strategy. As is standard, participants received AUD $15 (US $10.05) for lost work time. Funds were deposited into the inmates’ *buy-up* account.

### Ethics Approval

Human research ethics approval was obtained from Corrective Services New South Wales Ethics Committee (D20/1014950), the University of New South Wales Human Research Ethics Committee (HC210546), Justice Health and Forensic Mental Health Network Human Research Ethics Committee (2021/ETH11114), and The Aboriginal Health and Medical Research Council (1873/21) before the commencement of the study.

### Procedure

#### FG Approach and Schedule

We conducted 4 FGs, all of which were between 60 and 120 minutes long. All FGs were completed using internet-based audio-visual technology owing to the COVID-19 protocols. Sessions were led by the second author (AW), a neuropsychologist, and supported by RM and JH. FG questions were semistructured, and all 4 sessions followed a similar interview schedule. We were interested in game interventions but also more broadly in understanding people’s interaction with prison and past health services and their personal experiences with these services. After the objective of our study was discussed with participants, we showed a serious game demonstration (Graze Invaders; designed by AW, LM, and MK). This was used to provide a grounded example of what a serious game–based cognitive assessment might look like, in turn, setting some parameters for targeted design while still enabling participants to be open and creative [58] (refer to the study by Povey et al [21] for similar approach). After the general discussion about health and the demonstration, we asked targeted questions about what people thought about the serious game and then asked them to provide their general preferences for games and scrutinize the suitability (eg, logistically) of a game-based cognitive assessment in prison.

Example questions include the following:

1. Do you remember ever being asked questions about your memory or thinking while you have been in prison?
2. Have you ever had a memory or thinking test before coming to prison, maybe from a psychologist?
3. Before entering prison, did you ever use a computer or tablet?
4. Did you ever use a computer/phone/tablet to play games?
5. What kinds of extra things make you enjoy games more?
6. Do you think there would be any issues with you and other older adults in prisoner using this sort of brain game technology?
7. Is there anything else about using a serious game in prison you might like us to consider?

#### Data Analysis

This study used reflexive thematic analysis in which the active role of the researcher in producing knowledge is emphasized, with themes conceptualized as meaning-based patterns, rather than summaries of data [59]. Reflexive thematic analysis is compatible with our overarching philosophy of critical realism [60], which can be used to accurately explore the participant’s empirical world while engaging with underlying mechanisms and theories that can inform game design and development in the context of prison [44,61]. An abductive analytical approach was adopted to conduct this process. Abduction—also known as theoretical redescription—is a technique whereby empirical data are redescribed using theoretical concepts (in this case, SDT and game design research) [54,62]. Abduction has been defined as a process of “inference or thought operation, implying that a particular phenomenon or event is interpreted from a set of general ideas or concepts” [51]. Abduction raises the level of theoretical engagement beyond detailed description of the empirical entities but with an acknowledgment that the chosen theory is fallible [51]. This enables us to investigate participant observations in the context of latent, theoretical, and underlying mechanisms [51,53], which may be influencing game design preferences and motivations, while also ensuring that user observations and insights are still central to theme generation [56].

The analytical process followed the phases presented by Braun and Clarke [56,59,63]. Specifically, the first author transcribed and reread the interview transcripts and then coded and collated the interesting features of the data. Next, codes were added or updated deductively; that is, we explicitly analyzed our data through the lens of existing theory (namely, SDT). When we were satisfied with our coding, we used an abductive approach to combine and recontextualize existing codes. After coding and collating was completed, themes were drafted. These theory laden themes were analyzed to query any important underlying mechanisms, which may have been influencing the empirical observations. Finally, themes were organized and named in ways to appropriately portray the overall story of analysis. Using the process of abduction, all themes embody both the experiences of end users and relevant theories redescribing these observations in a context relevant to developing game design features [45].

### Validity

While generating themes, the rigor and quality of the analysis can be enhanced by considering the types of validity (descriptive, interpretive, and theoretical) by Maxwell [52] and broad indicators of validity such as empirical adequacy, ontological plausibility, and explanatory power [64]. Interviews were transcribed verbatim and then reread to check the accuracy of the transcription by the first author (RM), who also conducted the primary analysis. This addressed the questions regarding descriptive validity. The predetermined group design differences (women vs men; Aboriginal vs non-Aboriginal; and aged 50-64 years vs aged ≥65 years) in the FGs were used to establish consistency and key differences [49] in participant accounts from varied perspectives. The FG questions were partially informed by existing theoretical accounts on serious game design (eg, questions about game design features and typical duration of a game-based assessment); however, given the nature of FG sessions, general and open-ended questions were also asked, which enabled participants to discuss general ideas together and form intragroup consensus organically. The second author (AW) and senior author (JH) acted as *critical friends* [65] to the first author (RM), who provided critical dialogue and challenged the first author’s assumptions and explanations of phenomena at all stages of analysis (ie, initial coding, developing themes, and reviewing and naming themes). The use of the *critical friend* approach to thematic analysis underpinned by critical realism was conducted successfully by Goddard et al [44]. Finally, the applied basis for the research objectives (ie, to establish design preferences for an effective game-based assessment) produced clear practical utility [44,64] (this is discussed further in the *Limitations* section).

## Results

### Overview

Overall, 4 FGs with a total of 20 people were conducted with older adults (aged ≥50 years; aged ≥45 years for Aboriginal and Torres Strait Islander People) across 3 distinct prisons in New South Wales, Australia. Participants’ characteristics are presented in Table 1.

Overall, 3 distinct themes were generated. The first theme emphasizes the participants’ collective desire for an individually optimal level of difficulty in serious gameplay. The second theme raises the importance of avoiding immature and childlike gameplay features, as they can feel condescending to some older end users. The third theme highlights the strong user preference for meaningful choice and variety in any serious game–based cognitive assessment. According to SDT, the first theme relates to satisfying competence needs, whereas the second theme empathizes the need to avoid competence violations. The final theme is related to satisfying autonomy needs through meaningful choice.

**Table 1:** Participant characteristics.

Group; security classification and participants		Age (years)	Time served	Identified as Aboriginal	Sex
**FG^a^ 1; maximum security**					
	P1	52	<1 year	No	Female
	P2	54	<1 year	No	Female
	P3	49	<1 year	Yes	Female
	P4	51	<1 year	Yes	Female
	P5	53	<1 year	No	Female
	P6	54	<1 year	No	Female
**FG2; maximum security**					
	P1	49	>1 year	Yes	Male
	P2	57	>5 years	Yes	Male
	P3	61	>5 years	Yes	Male
	P4	63	>10 years	Yes	Male
**FG3; maximum security**					
	P1	66	>20 years	No	Male
	P2	47	>1 year	Yes	Male
	P3	52	>1 year	No	Male
	P4	64	>5 years	No	Male
	P5	60	>1 year	No	Male
**FG4; minimum/maximum security**					
	P1	72	>1 year	No	Male
	P2	76	>5 years	No	Male
	P3	82	>1 year	No	Male
	P4	90	>5 years	No	Male
	P5	69	>5 years	No	Male

^a^FG: focus group.

### Goldilocks—Getting Gameplay Difficulty Just Right Through Optimal Challenge

There was considerable diversity in background, time served, education level, experience with computers and technology, and health status across the FG participants. This contributed to a variety of different expectations and preferences for what a serious game ought to be and the level of complexity that should be incorporated into gameplay.

One of the older men from FG4 felt that a simplified approach would be the most suitable:

I was in rehab 40 years ago...A couple of the blokes complained that it was a complicated program for simple people. And it should have been a simple program for complicated people. So, keep it simple stupid.

Participant 3; FG4

In contrast, one of the women from FG1, who had plenty of previous experience with games, was interested in a challenging game-based assessment:

I’m a games freak! So, yeah, I play...Obviously [it’s good if] it gets harder, like to challenge us as you go, as you progress. Yeah, that would be good.

Participant 2; FG1

This desire for a challenge through gameplay emerged several times across FG1, FG2, and FG3:

You need a challenge to keep people interested.

Participant 5; FG3

However, FG4, which consisted of a group of older men (aged ≥65 years) residing in a Frail and Aged Unit, was keen to reiterate that whatever serious game was designed, it was best if it was “probably not too complicated” (participant 1; FG4).

One of the men in FG4 also added the following:

If you’re happy doing it, I don’t think it matters.

Participant 3; FG4

This highlighted that if the level of complexity in gameplay was appropriate, participants would be motivated to play it.

This desire for an optimal difficulty level [37,38] reflected a broad consensus across all groups that if the game provided a suitable challenge, it would be far more enjoyable.

When discussing a game he enjoyed, a participant from FG2 reflected the following:

Because you need skills to do it, it is pretty good.

Participant 2; FG2

What constitutes a challenge is likely to be different for each end user. For instance, the need for a technical and skill-based challenge by participant 2 in FG2 was not the preference of another member of FG2:

The other ones are too hard...I stay with Solitaire.

Participant 4; FG2

With this desire for different levels of difficulty, participants agreed about the need for challenge flexibility to ensure that game difficulty was suitable for each player:

As long as it challenges you, I’d probably stay with it.

Participant 5; FG3

...And if you can speed it up and slow it down, that gives people with no memory a better go at it. I can’t remember things very well, so that adjustment of the speed level is pretty good. That’s the only reason that I’d play that game.

Participant 4; FG3

According to SDT, participant observations and consensus about the need for optimal challenge reflect the need to satisfy the basic psychological need for competence [37,38]. In the context of games, optimal challenges can lead to a sense of efficacy and thus competence [39]. If a game is very easy, it becomes boring and reduces the likelihood of attentive engagement [66]:

If you lose interest early, it is doomed.

Participant 5; FG3

In contrast, if it is very hard, it can become frustrating and discouraging, thus also reducing the likelihood of motivation, engagement, and enjoyment:

My brain doesn’t work that quick.

Participant 4; FG2

Optimal challenge is mediated by an individual’s threshold for difficulty [39,66]. Difficulty creates the challenge that is essential for satisfaction of competence needs. However, the right level of difficulty will be vastly different across individuals, especially in the diverse prison context:

Maybe you can design the project that you’re doing to different levels of how people want to react to these things.

Participant 1; FG3

Given the participants’ need for optimal challenge, alongside the diversity in what challenge means for each person, a serious game–based assessment that can effectively differentiate game complexity appears to be important in the prison context to ensure that the challenge level is *just right* to address competence needs.

### Avoiding Childish Graphics—Gimmicky Gameplay Can Be Condescending

Participants from FG2 and FG3 agreed that if a game did not have modern graphics or if it appeared childish, it was not playable and was even condescending:

No disrespect for who designed that game [the demo shown to participants] but that’s a game for a 9-to-10-year-old...no offense but I couldn’t play that...

Participant 1; FG3

I’m 60 years old and I use the phone and the graphics on the phone are better than that. You can’t expect people to go back, to be honest.

Participant 1; FG3

It was clear that their expectations were for a game to feel more mature:

Your graphics are really bad [laughing]. Nintendo 64 was better than those graphics.

Participant 1; FG2

[The game needs to be] more realistic.

Participant 5; FG3

[And] more adult.

Participant 1; FG3

In contrast, the women from FG1 had a far more positive response to the serious game demonstration, with only 5% (1/20) of the participants finding the game to be problematic:

It’s giving me anxiety.

Participant 3; FG1

[This game demo is good] because memory tests aren’t usually fun.

Participant 5; FG1

I like it.

Participant 4; FG1

It’s cute.

Participant 2; FG1

When queried about their general game preferences, the women in FG1 reacted positively to the ability to choose a character, even a character of the opposite sex. A participant suggested the following:

That’s half the fun in it!!

Participant 4; FG1

The older men from the *aged ≥65 years* group were mostly indifferent to the game demonstration and did not comment about the graphics. They seemed to be more interested in functional issues connected to the game, such as whether it had audio and what the game was testing.

Although the openness to different game types varied across the groups, the strength of the aversion to *childish* games from several participants in FG2 and FG3 is important to be emphasized. Any game experience that makes a substantial portion of the target users feel condescended needs to be avoided:

I’d rather throw it out the window then just occupy my time with it. It has to be more than just the premise of being a game. In that regard, that’s condescending. I don’t consider myself unintelligent so, as someone said earlier, speak to me as an adult. Not as a ten-year-old kid. I understand there’s people who have learning problems and all the rest of it, but do I have to be spoken to in the same manner as them?

Participant 1; FG3

Games that felt childish and immature appeared to violate competence needs in FG2 and FG3 for some participants [37-39]. Although these negative responses were not evident in FG1 and FG4, the need to avoid competence violations is of high importance for any game-based assessment. This is especially true for a cognitive assessment game in the prison context, where people already report feeling overlooked and unheard [40-42].

Furthermore, if people are worried about their cognitive health before completing the game-based assessment, any risk of competence violations must be minimized, otherwise feelings of self-stigma or shame may be exacerbated [9-11]. A participant reflected the following:

Just a thought. When you’re planning for this...this is where psychs get it wrong...Say what you mean, mean what you say. We’re grown men. Don’t dabble around. Get to your point. Get your yes or no. Factor that in when you’re planning this stuff. Don’t treat humans like children. Nothing worse. It’s patronising and condescending. Makes you feel stupider than you know you already are.

Participant 2; FG3

Thus, the need to ensure that game-based assessments in prison environments do not feel overly childlike or immature appears to have vital importance:

As far as games and all that, we had the Atari and Mario Brothers and all that. We played it with the kids. It’s not just us playing. That was my involvement with games. But yeah, like the boys have said chess, backgammon, tiddlywinks, monopoly, we played all that. But I mean, games need to be more geared towards brain power, rather than just simplistic things like building blocks on top of other blocks like Tetris. But yeah, I got no problems with them.

Participant 1; FG3

Games that explicitly focus on *brain power*—such as quizzes, crosswords, and numeracy challenges—seem to be more aligned with the preferences and expectations of many participants in FG2 and FG3. In addition, there was no clear aversion to these types of *mature* games in FG1 and FG4, suggesting that they may be more appropriate for a wide range of older adults in prison.

### A Balanced Diet—Meaningful Choice and Variety Keeps Game-Based Assessments Fun

Given the subjective nature of game enjoyment, a variety of different, and at times, contrary, game preferences were highlighted.

For example, when people were asked if they liked gaming features such as earning badges for performance achievements, there was a spectrum of opinions:

No, I don’t want badges. I don’t wanna know that.

Participant 4; FG2

This contrasted with views such as the following:

Oh, I love winning. I’m a really good winner! Yeah. That’d be cool.

Participant 3; FG1

Game variety appeared to be a key preference for participants:

It doesn’t matter who you are, you can see on the screen, ok, I don’t know how to do that, but I’d like to know, so I’ll go and play that one...or, ok...I know how to do that, so I’ll go play that other one.

Participant 1; FG2

Yeah, you got to have a variety.

Participant 4; FG2

Participants were also quick to acknowledge and discuss the purpose of the game, that is, to test different types of cognitive functioning (reaction time, memory, executive ability, etc). By acknowledging the primary purpose of the game, participants saw the opportunity to create a variety of gamified tasks addressing different design appetites within the groups and within themselves:

Yeah, you could have mind games, where you gotta think about what you’re doing. And reactive games, where you gotta react to something and all that sort of thing...you could have literacy sort of games where you gotta think about what’s being written and other games where you’re using your reaction time and you’re thinking about what’s going on but you’re not reading it.

Participant 1; FG2

When brainstorming about game design preferences, a participant from FG2 even suggested the technique of game customization to avoid amotivation and maximize meaningful choice:

Ohh, I just come up with a thing you can do for what you need. If you got someone who is getting dementia and they are retired, and you knew what their trade was, with the trade they’d done for work, you could ask them things like...let’s say they done carpentry...Say, “what kind of tools would I need to build this kind of table and the process that I need to go through to start [to] build that table?” And you could see what their memory is from what they used to do. If you use to do something, your memory should recall, and you ask them something about that.

Participant 2; FG2

Another member of FG2 expanded upon this customization idea:

Going back to the building side of things, you could have a Mah-jong sort of game with building materials. Even engineering, you could do it with cars, make it like you gotta build a car or something.

Participant 1; FG2

Throughout the brainstorming conducted across a number of FGs, especially FG2, the game design technique of minigames emerged as a viable solution to address the need for variety and meaningful choice across the serious game.

When asked about the potential to use minigames, a participant positively reflected the following:

Like little minigames where, between what’s going on around them, they’re coming down like a map of different quests type thing? You’re progressing through, or something, and play a little mini game...then move on and play another mini game? Yeah, that sounds good. To break the attention up. Yeah, yeah, that that would work.

Participant 3; FG2

This preference for different minigames reinforces the appetite for meaningful choice, a critical element of autonomy in the context of game design [37-39]. According to SDT, autonomy in digital games involves interesting options and volitional engagement [67]. Although choice and variety are critical design elements in most games, the strong desire for these elements by participants is important to be highlighted in the context of prison—an environment where autonomy is often thwarted [41].

## Discussion

### Principal Findings

This study aimed to qualitatively examine the design preferences for a serious game–based cognitive assessment among older adults in prison. Using an abductive approach underpinned by critical realism, we generated 3 distinct themes. They were (1) Goldilocks—getting gameplay difficulty *just right* through optimal challenge, (2) avoiding childish graphics—gimmicky gameplay can be condescending, and (3) a balanced diet—meaningful choice and variety keeps game-based assessments fun. These themes provide novel insights into the game design preferences of older adults in prison, whose voices have been seldom heard in the development of serious games so far. In addition, the production of these themes in the context of SDT and game design research should, we hope, provide a useful and actionable base for serious game designers to develop appropriate cognitive games that are acceptable and motivating for older adults within the unique prison context.

Within theme 1, it was clear that the competence needs for an individually optimal level of difficulty was a priority across all FGs. Regarding game design, this can be addressed by developers through dynamic game balancing [66,68]. Dynamic game balancing is a popular game design technique that overcomes variability in individual difficulty thresholds through real-time adjustment of game parameters, so that task difficulty adapts and eventually aligns with a player’s ability [66]. This ensures that competence can be satisfied for a variety of users through optimal challenge [69]. The goal is to keep the user interested from beginning to the end. Dynamic game balancing is associated with high levels of user satisfaction [70] and feelings of competence, which predicates high levels of motivation and enjoyment [69]. The use of dynamic game balancing for serious game–based cognitive assessments is an emerging area, and potential complexities such as maintaining psychometric validity are yet to be fully considered [66]. Nonetheless, the technique appears to be theoretically capable of addressing the competence needs for optimal challenge highlighted by participants.

Theme 2 reflected the need to avoid competence violations by minimizing childlike game features, which made some participants feel condescended. In the context of SDT, game features offering opportunities for mastery that provide optimal challenges can satisfy competence needs by helping a player feel a sense of accomplishment and control [38]. However, the opposite can also be true. When people are tasked with playing a game that they believe is not suitable for them or does not align with their self-perceived level of capability, it can become boring, frustrating, and even insulting. This is an especially important consideration in the context of prison, where the need for competence is often thwarted, and people often distrust the prison system and report feeling disrespected by health and corrective professionals [71]. A noteworthy minority of our study participants highlighted that childish graphics made them feel condescended, and some members of our FGs appeared to be frustrated by the idea of playing a childish game for the purpose of cognitive assessment. We suggest that this frustration stems, at least in part, from a misalignment between some participants’ self-perceived level of capability and the perceived inability of a game that looks and feels childish to meet this capability. We further suggest that this frustration, a common and natural response to perceiving a game as incompatible with one’s intellectual capacity, can be exacerbated by the competence-thwarting prison environment, in which control is already difficult to achieve [72]. Thus, avoiding games that feel similar to *kids’ games* and designing features that are more *adult* and *realistic*, which are *geared toward brain power* appear to be important to avoid competence violations and, more broadly, guard against overly negative responses to serious game–based assessments in prison.

Theme 3 highlighted the strong user preference for choice and variety, both of which satisfy the need for autonomy and can enhance intrinsic motivation [37,38,67]. Although the positive effects of choice can be complex and depend on a variety of factors (eg, cultural background), multiple studies have shown that choice has a beneficial effect on motivational outcomes such as effort and task performance [41,73]. Autonomy satisfaction has also been associated with better quality of life in prison [41]. However, both autonomy [74,75] and choice [76] are often difficult to attain in prison [41]. Therefore, ensuring that games are autonomy-focused through *meaningful choice* when they are restricted in real life may be particularly beneficial for this group. Minigames are an effective game technique to maximize choice and variety [38,39] and, as highlighted by participants, align well with the objectives of cognitive assessment. Thus, designing minigames with emphasis on user control appears to be a suitable way to maximize in-game autonomy for people from marginalized groups who may struggle to achieve autonomy in other aspects of their lives.

### Limitations

This study has some important limitations. First, this study purposefully sampled end users from 3 different prisons in New South Wales, Australia. Although attempts were made to select a distinct variety of older adults in prison and in turn understand empirical observations in terms of prominent theories or underlying structures, the themes generated are not necessarily generalizable to other locations or user groups. Second, all FGs were monitored by a correctional officer owing to security protocols. This power imbalance may have affected the responses given by participants, particularly those who are highly reliant on staff, such as those in the Frail and Aged Unit. However, no evidence of this was observed during the FGs. Third, the method of analysis used in this study and our interpretation of data are underpinned by the philosophy of critical realism. It is entirely possible that a researcher informed by a different philosophical approach would interpret the empirical observations differently and thus make different truth claims. Similarly, results are reported using the technique of abduction. We have made attempts to be clear and transparent about our theory-focused interpretations and recontextualization of the data when producing our results. However, it is critical to acknowledge that our interpretations are inevitably based on our own beliefs and objectives. In addition, our chosen theories are fallible. To overcome this, we attempted to reduce biases and improve validity (refer to the *Validity* section); however, these attempts are not perfect. Moreover, they are not intended to remove the researcher completely from the qualitative process. With this in mind, it should be noted that the authors of this paper are focused on building a serious game for cognitive assessment that is suitable for use by diverse older adults in prison. We want the game to be successful and have likely interpreted participant observations and preferences to ask *how to make a serious game work* rather than *whether serious games work?* This does not mean we are certain that a game will be valid, feasible, or acceptable in prison. However, we certainly hope it will be, and this desire to design and develop a suitable serious game has informed this study.

### Practical and Research Implications

A serious game–based assessment provides a novel challenge for game designers and developers. This is because the intended application of a serious game assessment is likely to be a relatively short (eg, 10-20 minutes) user experience, which is only *played* by users sporadically. The implication is that traditional game design focusing on intrinsically motivating features such as narrative and social connectedness may not be feasible or desirable. For instance, a complex and value-based storyline, with unique characters and quests, is likely very complex for an assessment that someone is going to undertake irregularly for 10 to 20 minutes. This tension has not been given much attention in the serious game literature from a user design perspective. Most of the previous studies on user preferences of cognitive game design has highlighted the importance of embedding intrinsically motivating game features without considering the complexity of embedding these features into a very short and sporadically played game-based assessment [77]. These traditional features may be desirable in long-form games focused on cognitive training or rehabilitation but are less suitable for the narrow and short objectives of a cognitive assessment. In contrast, other attempts to make game-based cognitive assessments have gamified traditional tasks in very basic ways. Although this may be more suitable to ensure psychometric validity, this process risks not being particularly engaging or fun. For instance, some previous attempts to digitize cognitive tasks or introduce simple gamification have been unsuccessful, with users reporting that, similar to traditional tasks, the experiences were tedious and boring [3,20,78,79].

Our study was guided in part by the need to address this tension. We were open with our participants about the typical length of serious games and the intended purpose of a gamified cognitive assessment (ie, to detect cognitive decline or dementia). This enabled users to provide practical design insights that reflect the unique parameters of serious game–based cognitive assessment. As such, the themes generated are consistent with the reality of serious game–based design and conform to the nuances of gamifying short and sporadic assessments while still highlighting game design features that are immersive, motivating, and enjoyable to our distinct user group. In this way, optimizing the challenge by balancing the difficulty level, avoiding childish features, and maximizing meaningful choice through minigames are unique user-generated design preferences suited to addressing user needs within a short and potentially infrequent serious game–based assessment in the prison setting.

## Abbreviations

FG: focus group
SDT: self-determination theory
